# Femoral artery thrombosis after internal fixation of a transverse acetabular fracture in a patient with osteogenesis imperfecta type I

**DOI:** 10.1186/1754-9493-2-1

**Published:** 2008-01-14

**Authors:** Navid M Ziran, Jeffrey L Johnson, Steven J Morgan, Wade R Smith

**Affiliations:** 1 Department of Orthopaedics, Rocky Mountain Regional Trauma Center, Denver Health Medical Center, Denver, Colorado, USA; 2 Department of Surgery, Rocky Mountain Regional Trauma Center, Denver Health Medical Center, Denver, Colorado, USA

## Abstract

Osteogenesis imperfecta is a genetic disorder characterized by increased susceptibility to fractures and vascular injuries due to connective tissue fragility. In this case report, we present a patient with osteogenesis imperfecta type I who sustained a transverse fracture of the right acetabulum while transferring from bed to chair. The fracture was repaired through an ilioinguinal approach. During the surgery, an iatrogenic injury to the femoral artery and vein occurred. This intraoperative complication was salvaged by immediate vascular repair. We discuss the possible causes of iatrogenic vascular injuries in patients with osteogenesis imperfecta. Orthopaedic surgeons should be aware of this potentially devastating complication in this particular patient cohort.

## Background

The ilioinguinal approach is frequently used in orthopaedic pelvic surgery to repair the anterior column, the anterior wall, and transverse fractures of the acetabulum [1]. This approach has a lower incidence of heterotopic ossification and does not violate the abductors – thus, making it an appealing approach for the treatment of these fractures. The inherent risks involved in this approach are damage to the lateral femoral cutaneous nerve, injury to the femoral vessels, bleeding from damage to the *corona mortis*, inguinal hernia, hematoma, infection, and problems with lymphatic drainage. In this report, we present a patient with Osteogenesis Imperfecta type I who sustained damage to the femoral artery and vein during open reduction internal fixation of a transverse acetabular fracture via the ilioinguinal approach.

## Case Report

**Figure 1:**
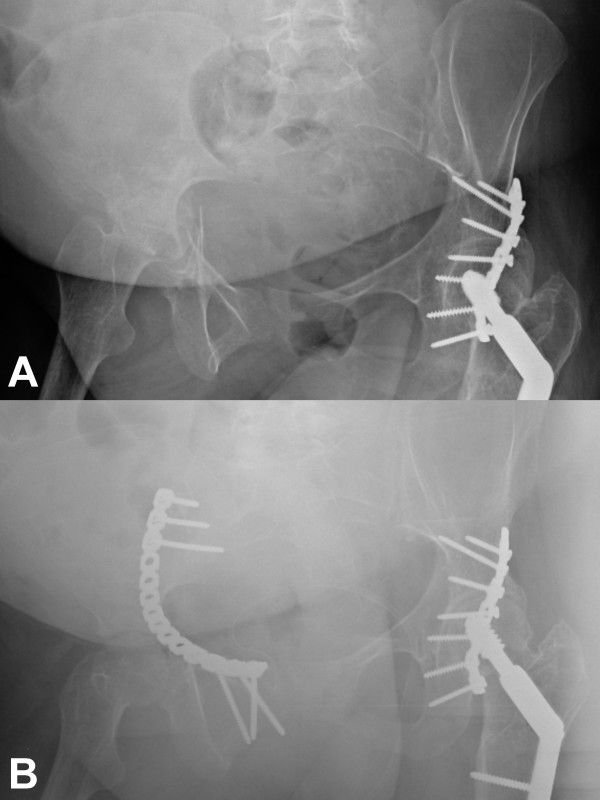
AP pelvis views demonstrating a transverse fracture of the right acetabulum pre-operatively (A) and post-operatively (B). The hardware on the left is from prior fixation.

**Figure 2:**
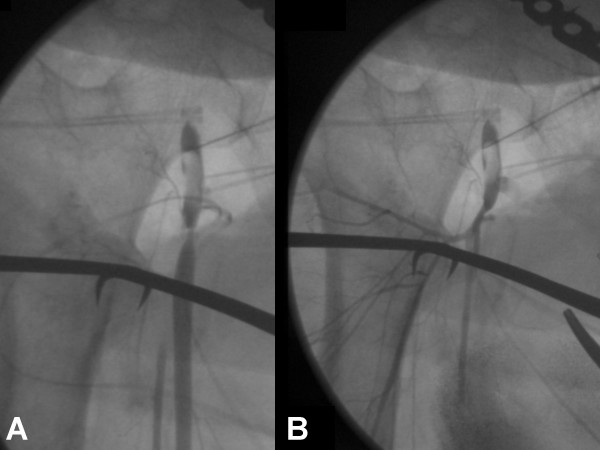
Intra-operative angiogram demonstrating occlusion of the right superficial femoral artery (A). Angiogram post-thrombectomy which demonstrates restored flow through the right superficial femoral artery (B).

The patient is a 25 year old female with Osteogenesis Imperfecta type I, and she is a limited household ambulator with numerous orthopaedic procedures in the past. She presented to our Emergency Department after being transferred from a bed to a chair. She heard a pop in her right hemipelvis and complained of right hip pain and left wrist pain. Upon evaluation, she was found to have a significantly comminuted right transverse acetabulum fracture (Figure 1a) and a left distal radius fracture. Two days after presentation, she underwent open reduction internal fixation of the right acetabulum fracture via a standard ilioinguinal approach (Figure 1b). During the initial dissection, the femoral vessels were noted to have minimal perivascular tissue. After exposure, the comminuted fracture was reduced using an offset clamp. A 14-hole reconstruction plate was placed along the pelvic brim along the length of the anterior column. During placement of screws in the pubis with retraction on the femoral vessels, it was noted that there was significant venous bleeding from the vascular bundle. The greater saphenous vein and the femoral vein had been partially lacerated by the offset clamp. This laceration was only identified as the offset clamp was removed since the clamp was providing some tension and pressure on the laceration. Hemostasis was achieved by digital pressure. Acute care surgery was notified and repaired the vein with 6-0 monofilament suture without complication. The rest of the screws were then placed in the plate, two self-suction drains were placed, and the wound was closed. After the drapes were removed, the patient's right leg had a mottled blue appearance, was cold to touch compared to the contralateral side, and had no palpable pulses. Venous doppler signals were obtained for both the posterior tibial vein and dorsalis pedis, but no triphasic waveforms were present. Again, acute care surgery was notified. The middle window of the approach was opened. The femoral artery had a contusion and had a palpable pulse only proximal to this contused area. An arteriotomy was done with angiography which revealed a thrombus with significant occlusion of the femoral artery. A thrombectomy was performed both proximal and distal to the arteriotomy with restoration of angiographic flow distally (Figure 2a and 2b). The artery was repaired with monofilament suture, and the wound was closed. After the procedure, the patient had palpable pulses and satisfactory triphasic waveforms of both the dorsalis pedis and posterior tibial artery. Due to possible pulmonary overload from significant intraoperative transfusion requirements, the patient remained intubated and was taken to the surgical intensive care unit. Post-operatively, the patient was placed on a heparin drip and Coumadin for 2 weeks. She underwent wound exploration with vacuum-assisted closure (VAC) placement due to concern of poor tissue closure with a risk of possible infection. She underwent a VAC replacement during the hospital course and was sent home 2 weeks after the initial operation with a VAC as definitive treatment.

## Discussion

There has been a prior report of a femoral artery thrombus during an ilioinguinal approach for a transverse acetabular fracture [2], but none have been reported in patients with Osteogenesis Imperfecta (OI). OI is an inherited congenital condition due to mutations in type I procollagen type I (COL1A1 and COL1A2). There are four major types: I = mild, II = very severe, III = severe, IV = undefined [3]. Patients will usually present with fractures in the mildest form, but due to the qualitative and quantitative defect in type I collagen, patients may also have blue sclera, problems with dentinogenesis, hearing loss, kyphoscoliosis, and easy bruising. Vascular fragility has not classically been associated with osteogenesis imperfecta, although there have been numerous reports of vascular injury either from mild head trauma [4-6] or during surgical intervention [7]. This may be in part due to the fragility of the connective tissue, coagulation abnormalities, or poor platelet aggregation in these patients but has also been attributed to platelet abnormalities [8]. In our case, while we cannot attribute vascular injury solely to the patient's genetic condition, we do feel that the patient's injury was out of proportion to the trauma sustained since care was taken to avoid excessive retraction or trauma to the femoral bundle. We did not feel that the injury was immediate since after placement of the clamp, the bundle was not entrapped nor was there any extravasation of blood. Rather, the injury occurred after prolonged retraction while the offset clamp was in place and during screw placement. Once the clamp was removed, the bleeding was more obvious, and the laceration to the greater saphenous vein was identified. Thus, it is likely that the patient's condition was a result of a combination of vascular fragility in combination with either prolonged retraction or excessive digital pressure during venous hemostasis. During digital pressure on the vein, damage may have also occurred to the femoral artery – which, due to the patient's OI, may have been predisposed to injury. As a result of vessel wall fragility and injury, platelet aggregation occurred with activation of the tissue factor coagulation cascade and subsequent clot formation. Because of the lack of definitive qualitative platelet and coagulation factor studies in OI patients, there may have been other unknown factors contributing to thrombus formation. Thus, in OI patients with acetabular or pelvic fractures, we would recommend avoiding *any *retraction on the neurovascular bundle. Another means of avoiding injury during the ilioinguinal approach is to only open the lateral and medial windows, thus avoiding all dissection of the neurovascular bundle. Reduction of the anterior column or transverse fractures can be attained by a combination of indirect/direct reduction and 3.5 mm reconstruction plate fixation using the lateral and medial windows. Finally, if the joint is congruent and the fractures are not significantly displaced, consideration should also be given to limited open reduction and/or closed reduction with percutaneous screw fixation [9]. In conclusion, extreme care should be taken to avoid iatrogenic fracture and/or vascular injury in OI patients during acetabular/pelvis fracture surgery; less invasive means of fixation should be considered in this patient cohort.

## Abbreviations

OI: Osteogenesis imperfecta; VAC: Vacuum associated closure

## Declaration of Competing interests

The author(s) declare that they have no competing interests.

## Authors' contributions

NZ, JJ, SM and WS all contributed to the scientific content of the manuscript.
